# Periodically spilled-oil input as a trigger to stimulate the development of hydrocarbon-degrading consortia in a beach ecosystem

**DOI:** 10.1038/s41598-017-12820-7

**Published:** 2017-09-29

**Authors:** Kai Zhang, Yongge Sun, Zhisong Cui, Di Yu, Li Zheng, Peng Liu, Zhenmei Lv

**Affiliations:** 1 Environmental and Biogeochemical Institute (eBig), School of Earth Science, Zhejiang University, Hangzhou, Zhejiang, 310027 China; 2 The First Institute of Oceanography, SOA, Qingdao, Shandong 266061 China; 3 Wuxi Research Institute of Petroleum Geology, SINOPEC, Wuxi, Jiangsu 214126 China; 4 State Key Laboratory of Shale Oil and Gas Enrichment Mechanisms and Effective Development, Wuxi, Jiangsu 214126 China; 5 School of Life Science, Zhejiang University, Hangzhou, Zhejiang, 310058 China

## Abstract

In this study, time-series samples were taken from a gravel beach to ascertain whether a periodic oil input induced by tidal action at the early stage of an oil spill can be a trigger to stimulate the development of hydrocarbon-degrading bacteria under natural *in situ* attenuation. High-throughput sequencing shows that the microbial community in beach sediments is characterized by the enrichment of hydrocarbon-degrading bacteria, including *Alcanivorax*, *Dietzia*, and *Marinobacter*. Accompanying the periodic floating-oil input, dynamic successions of microbial communities and corresponding fluctuations in functional genes (*alk*B and RDH) are clearly indicated in a time sequence, which keeps pace with the ongoing biodegradation of the spilled oil. The microbial succession that accompanies tidal action could benefit from the enhanced exchange of oxygen and nutrients; however, regular inputs of floating oil can be a trigger to stimulate an *in situ* “seed bank” of hydrocarbon-degrading bacteria. This leads to the continued blooming of hydrocarbon-degrading consortia in beach ecosystems. The results provide new insights into the beach microbial community structure and function in response to oil spills.

## Introduction

The marine coastal environment is highly susceptible to oil spills induced by the expansion of offshore oil and gas exploration and transportation. Despite aggressive efforts to remove oil slicks as an emergency response, a large portion of lingering oils still wash in and are eventually trapped on the shoreline^1–3^, thereby causing great public and environmental concerns^4^.

It has long been recognized that the ultimate fate of oil patches deposited on beaches strongly depend on the natural attenuation capacity of the indigenous microbes^2,5^, although degradation rates are highly variable and result in environmental constraints. Previous studies demonstrated that biodegradation is mainly controlled by complex interactions between the microbial community and local environmental complexity (*e*.*g*., bio-availability of residue oil, temperature, nutrients, the presence of oxygen, etc.)^6–8^. Therefore, understanding the interactions between the microbial community and physiochemical properties is a prerequisite for effective remediation strategies for spilled oil.

Compared to mangroves, salt marshes, intertidal sediments and even permeable sands, exposed coarse beaches usually represent a rough habitat for self-remediation, due to their low biodiversity and low productivity^9,10^. However, beach sediment has been recognized as an important site of nutrient exchange with water, and spilled oil may have a potential influence on nutrient transformation^11,12^. One famous example is the Exxon Valdez oil spill. To accelerate the degradation rate of residue oil in beaches, large-scale field trials with added fertilizer were conducted after an oil spill accident^13,14^. However, a recent study by Malakoff^4^ revealed that lingering oil persisted on the beach 25 years later. Even worse, trapped oil appeared to be fresh in the sediments, suggesting that biodegradation in the beach ecosystem is more complicated than expected^15^.

Coarse beaches (pebble/cobble) have been recognized for a high rate of oil degradation (natural attenuation) since the 1970s due to direct exposure of oil residue to oxygen and sunlight^5,16^. Photooxidation, biodegradation, evaporation, dissolution and strong shoreline energy were presumed to be responsible for accelerating the oil weathering process in a beach system. The oil biodegradation rate strongly depends on environmental factors that may stimulate the development of *in situ* hydrocarbon-degrading consortia. This phenomenon was recently hypothesized as an “autoinoculation effect” by Valentine *et al*.^17^ in the investigation of Deepwater Horizon oil spill in the Gulf of Mexico. A single oil-polluted beach is usually characterized by enrichment in hydrocarbon-degrading bacteria, including many members of *Alpha-* and *Gammaproteobacteria* ^18,19^ and dynamic phylogenetic shifts of ammonia-oxidizing archaea (AOA) involved in nutrient cycles after the spill^20^. Theoretically, an oil polluted beach can be a natural laboratory to test the “autoinoculation effect” hypothesis due to the pulsed oil input and nutrient and oxygen exchange induced by periodic tidal action. Previous studies mainly explored whether the microbial community structure shifts in an oil-polluted beach after the accident; indeed, no significant change was observed at the class level to date^18^. Therefore, if the “autoinoculation effect” hypothesis is correct, the response of the microbial community in a polluted beach over time can be easily monitored and captured. Such a study can further probe how it interacts with or benefits from periodic oil input induced by tidal action during the early stage of an oil spill, especially for the microbial dynamic pattern and its drivers.

**Figure 1:**
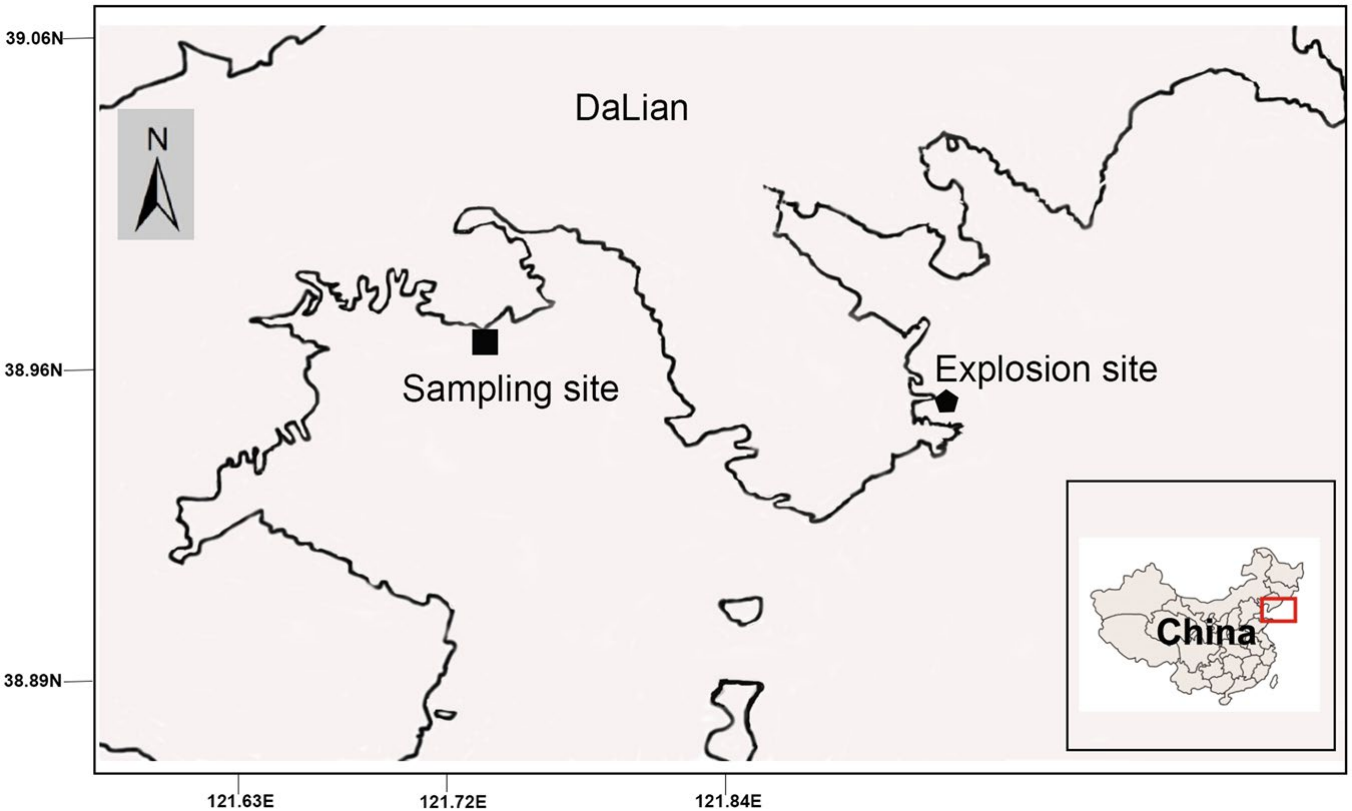
Location map showing sampling site in the Dalian Bay, China. The sampling site was shown for square, and explosion site was shown for pentagon. The figure map was generated by using software Google Earth (open-access version: 7.1.5.1557)(Data SIO, NOAA, U.S. NAVY, NGA, GEBCO, Image@2017 TerraMetrics) and the tracing drafted by using software CorelDRAW (Graphics Suite × 6, source ID: 017002) (http://www.coreldraw.com/en/product/graphic-design-software). The sampling sites were located by using Global Positioning System (GPS).

The Xingang oil spill occurred on July 16^th^, 2010, North China (Fig. 1). The disaster released approximately 100,000 cubic meters of crude oil into Dalian Bay, representing the largest oil spill accident to date in China. Although extensive clean-up efforts were undertaken by the government to reduce the damage, both the seawater and shoreline along Dalian Bay were severely polluted with oil^21^. This provides an excellent opportunity to investigate the microbial succession pattern and its controlling factors in a beach ecosystem with tidal influence over time and to verify the “autoinoculation effect” hypothesis. The objectives of this study were (1) to probe the response of the microbial community in beach sediment to periodic oil input due to tidal action and (2) to verify the “autoinoculation effect” from mixing and circulation processes in beach sediment.

## Results

### Chemical composition of residue oil over time

**Figure 2:**
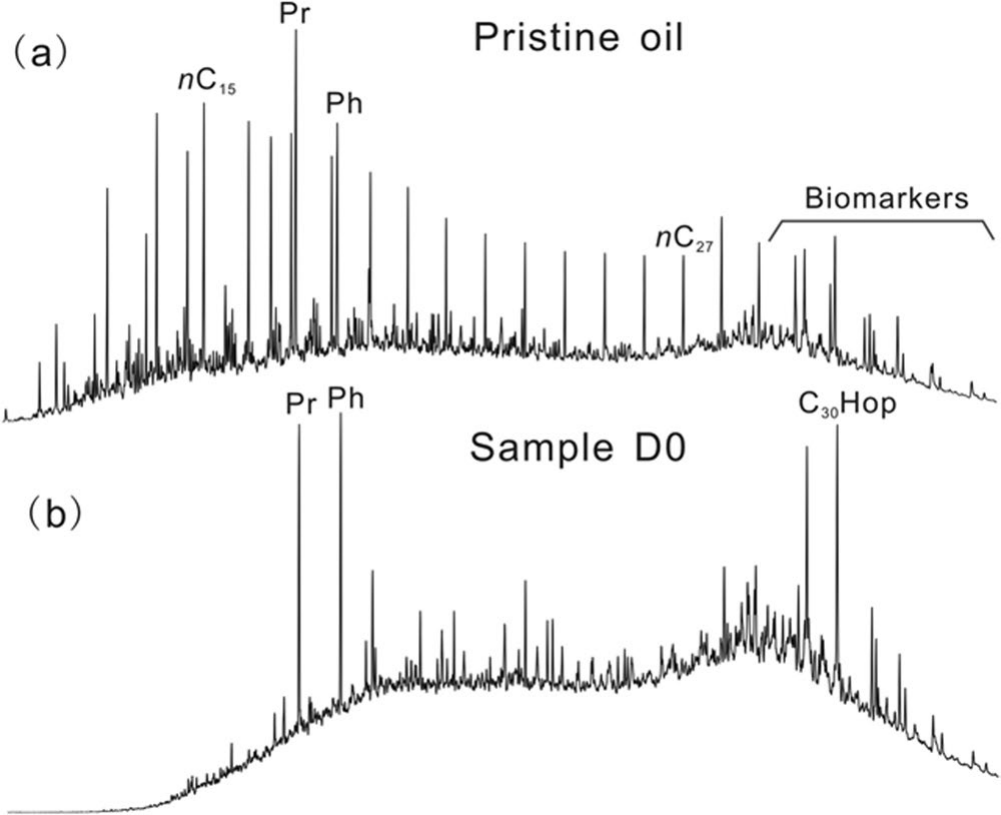
Gas chromatograms of aliphatic hydrocarbons separated from pristine oil (**a**) and first collected residue oil D0 in gravel beach (**b**). Pr = Pristane, Ph = Phytane, *n-*C_15_ = normal alkane with carbon number of 15, *n-*C_27_ = normal alkane with carbon number of 27, C_30_Hop = C_30_ 17α(H)21β (H)Hopane.

The originally spilled oil is heavy oil with an API (The American Petroleum Institute) Gravity of 16°, although light oil is added to increase its mobility. Therefore, the whole oil gas chromatogram displays the full series of normal alkanes ranging from *n*-C_7_ to *n*-C_35_ but is still characterized by significant unresolved complex mixtures (UCM), which is a typical indicator of heavy oil (Fig. 2a). However, the first sample (D0) collected from the gravel beach 34 days after the spill accident shows the typical characteristics of severely degraded oil, with almost total removal of *n*-alkanes, enrichment in branched and cyclic alkanes, and a high UCM (Fig. 2b). Thereafter, although the gravel beach is periodically affected by tidal action with fresh inputs of spilled oil, the residue oils, depending on the time sequence, exhibited full depletion of *n*-alkanes, a decreasing trend for isoprenoids, and no discernible changes in saturated biomarker distributions (*e*.*g*., steranes, hopanes; Supplementary Fig. S1). This suggests that the oil patch from the beach had undergone a variety of natural weathering processes, including emulsification, dispersion, dissolution, volatilization, photooxidation and biodegradation while floating on the sea surface, leaving behind viscous residues such as tar balls and other recalcitrant compounds. Photooxidation and biodegradation could be the main weathering processes accounting for the formation of severely degraded oil with high UCM. Although the pattern of oil weathering due to photooxidation, in which aromatic hydrocarbons are more susceptible than aliphatic hydrocarbons, was generally consistent across different conditions^22,23^, it is difficult to differentiate this from biodegradation with conventional gas chromatography-mass spectrometry (GC-MS) techniques, due to transformation to polar species^24^. Recent studies with Fourier transform ion cyclotron resonance mass spectrometry (FT-ICR MS) demonstrated a decreased abundance of pyridinic nitrogen (N_1_ species), concurrent with an increased abundance of N_1_O_x_ and a predominance of high-order oxygen species (O_x_) induced by oxidation of petroleum compounds after sunlight exposure^25,26^. We analyzed polar fractions at the molecular level using FT-ICR MS. The results show no significant change in N_1_ species, N_1_O_x_ species, and high-order oxygen species during the time sequence (Supplementary Fig. S2), suggesting that biodegradation plays a major role in the formation of severely degraded oil here. One reasonable explanation is that residual oil on beaches was entrapped by sand, which could either block direct sunlight or form large oil agglomerates with very little exposed surface area. These processes may interfere with weathering reactions^27^.

Overall, the time sequence of the sample series identified by days can be approximately equivalent to the biodegradation indices Peters & Modowan Scal (PM) = 3–5 for petroleum geochemistry^28^. Aliphatic and aromatic fractions (compound-grouped fractions) generally show a decreasing trend with time, from 32.89% to 14.57% and 28.95% to 19.26%, respectively (Supplementary Tab. S1). Quantitative analyses of pristane, phytane, trimethylnaphthalenes and phenanthrenes also reveal concentration changes during the course of natural biodegradation. Pristane and phytane are quickly biodegraded, and their concentrations decrease from 0.67 μg/mg.oil to 0.03 μg/mg.oil and from 0.60 μg/mg.oil to 0.02 μg/mg.oil, respectively (Table 1). The concentrations of trimethylnaphtahlenes and phenanthrenes decreased from 0.11 μg/mg.oil to 0.002 μg/mg.oil and from 0.19 μg/mg.oil to 0.004 μg/mg.oil, respectively. The changes in the chemical composition of residue oil clearly suggest ongoing biodegradation in beach sediment.

**Table 1:** Concentration dynamics of isoprenoids, trimethylnaphthalenes, and phenanthrenes in residue oils accompanying biodegradation course (μg/mg.oil).

Samples	Isoprenoids^1^			Trimethylnaphthalenes^2^									Phenanthrenes^3^					
	Pr	Ph	Sum(Pr + Ph)	TrMN-1	TrMN-2	TrMN-3	TrMN-4	TrMN-5	TrMN-6	TrMN-7	TrMN-8	Sum(TrMN)	Phe	3-MP	2-MP	9-MP	1-MP	Sum(Phe)
D0	0.6677	0.5911	1.2588	0.0102	0.0232	0.0194	0.0135	0.0038	0.0227	0.0034	0.0122	0.1084	0.0307	0.0306	0.0371	0.0483	0.0423	0.1890
D3	0.0910	0.0848	0.1758	0.0045	0.0108	0.0102	0.0052	0.0012	0.0127	0.0017	0.0068	0.0531	0.0186	0.0222	0.0322	0.0412	0.0369	0.1511
D12	0.4661	0.4177	0.8832	0.0049	0.0127	0.0128	0.0062	0.0018	0.0140	0.0025	0.0070	0.0619	0.0099	0.0100	0.0113	0.0181	0.0222	0.0715
D15	0.0444	0.0416	0.0860	0.0002	0.0006	0.0010	0.0002	0.0001	0.0007	0.0015	0.0002	0.0045	0.0016	0.0006	0.0003	0.0007	0.0008	0.0040
D22	0.0272	0.0197	0.0469	0.0002	0.0004	0.0003	0.0002	0.0001	0.0003	0.0001	0.0002	0.0018	0.0012	0.0004	0.0007	0.0007	0.0006	0.0036
D32	0.0467	0.0648	0.1115	0.0015	0.0031	0.0029	0.0019	0.0005	0.0037	0.0007	0.0019	0.0162	0.0071	0.0089	0.0120	0.0173	0.0156	0.0609
D42	0.0835	0.0678	0.1513	0.0014	0.0028	0.0023	0.0019	0.0005	0.0029	0.0004	0.0014	0.0136	0.0067	0.0041	0.0051	0.0067	0.0059	0.0285
D52	0.1322	0.1623	0.2945	0.0010	0.0025	0.0027	0.0011	0.0003	0.0030	0.0009	0.0015	0.0130	0.0038	0.0039	0.0049	0.0079	0.0095	0.0300

(1) Pr = Pristane, Ph = Phytane.

(2) TrMN-1 = 1,3,7-trimethylnaphthalene, TrMN-2 = 1,3,6-trimethylnaphthalene, TrMN-3 = 1,3,5-trimethylnaphthalene + 1,4,6-trimethylnaphthalene, TrMN-4 = 2,3,6-trimethylnaphthalene, TrMN-5 = 1,2,7-trimrthylnaphthalene, TrMN-6 = 1,6,7-trimethylnaphthalene + 1,2,6-trimethylnaphthalene, TrMN-7 = 1,2,4-trimethylnaphthalene, TrMN-8 = 1,2,5-trimethylnaphthalene.

(3) Phe = Phenanthrene, 3-MP = 3-methylphenanthrene, 2-MP = 2-methylphenanthrene, 9-MP = 9-methylphenanthrene, 1-MP = 1-methylphenanthrene.

### Microbial community in sediment from the beach

**Figure 3:**
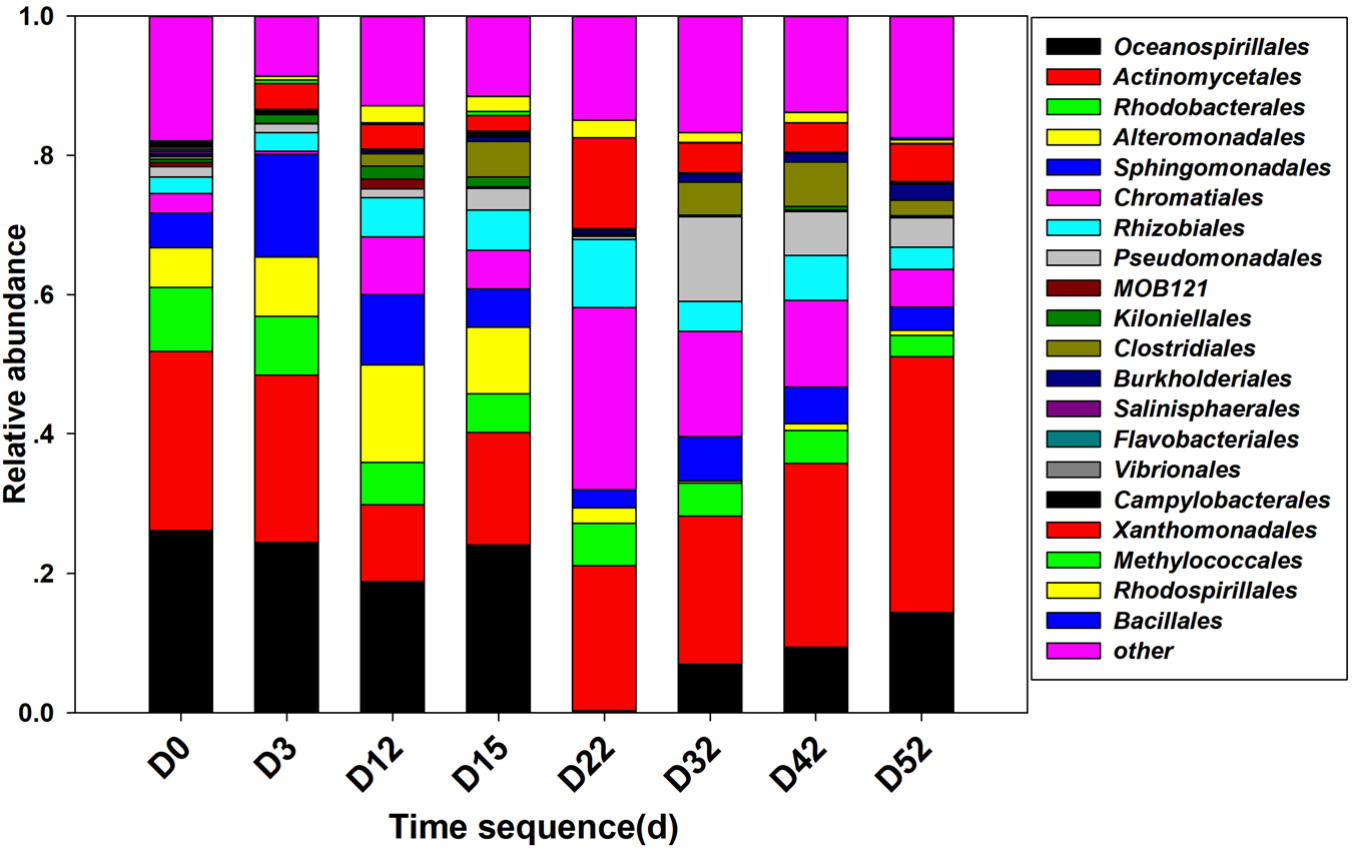
Phylogenetic shifts of dominant microbial taxa at order level in sediments as revealed by Miseq sequence. The relative abundance is presented in terms of percentage in total effective bacterial sequences for each sample.

**Figure 4:**
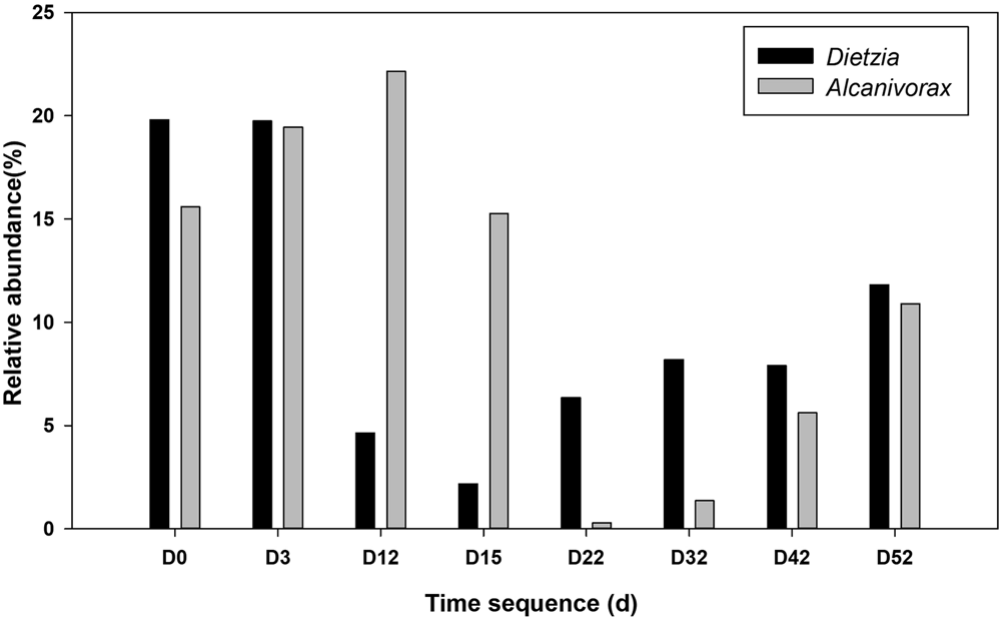
Relative abundance of *Alcanivorax* and *Dietzia* in sediments during the sampling campaign.

**Figure 5:**
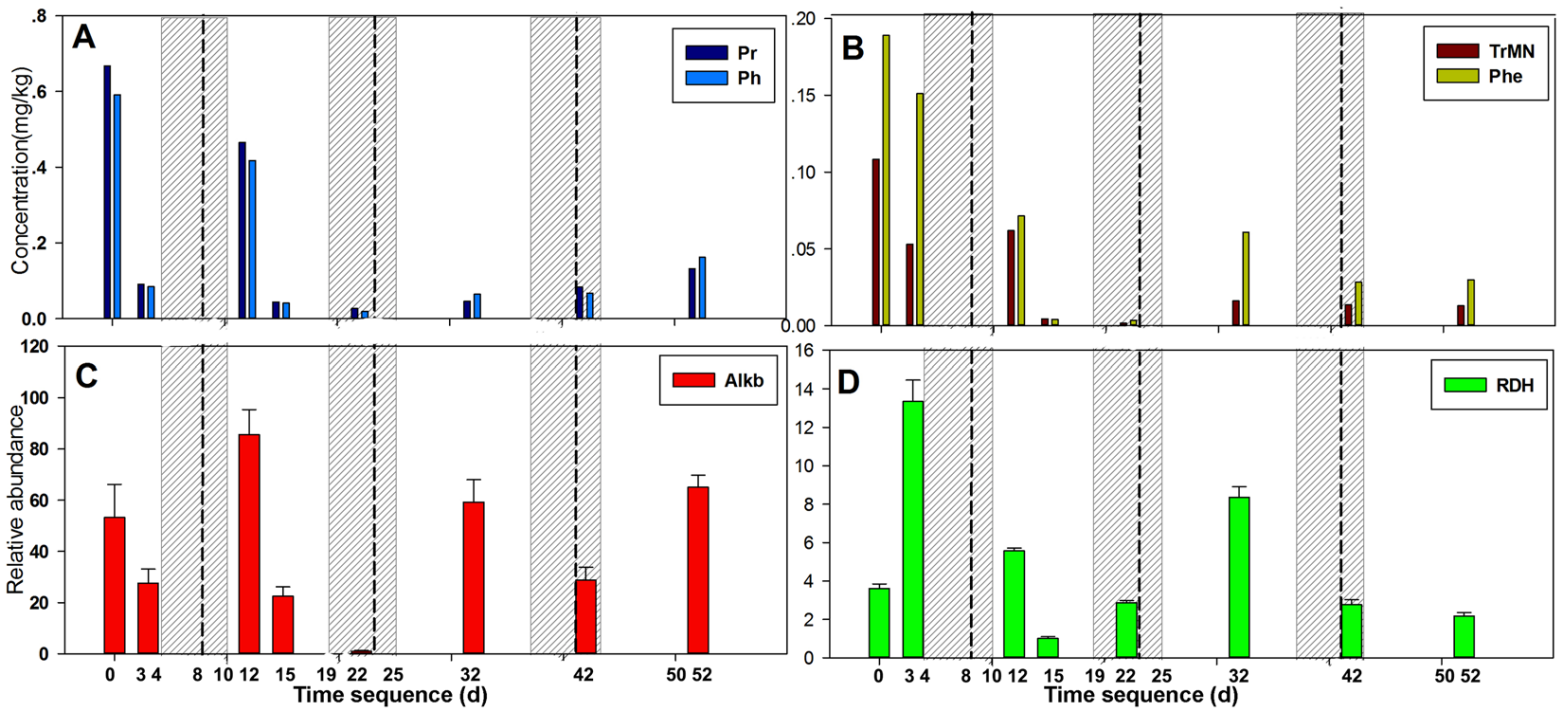
Time-series shifts of selected geochemical and biological parameters during the sampling campaign. A = concentrations of Pristane and Phytane; B = concentrations of trimethylnaphthalene and Phenanthrene; C = relative abundance of alkane 1-monooxygenasegene (*alkB*) gene; D = relative abundance of Ring-hydroxylating dioxygenase (RDH) gene. The dashed lines represent the maximum tidal action at every tide period and shadow area for the time duration of every tide period.

The Miseq high-throughput sequencing approach is used to obtain information about the beach microbial community structure. Specifically, sample D22 was sent for Roche 454 analysis to acquire deep insight into the community. The Roche 454 measurement reveals that *Proteobacteria* is the most abundant phylum in the sample, accounting for up to 81% of the total effective bacterial sequences, whereas *Bacteroidetes* and *Acidobacteria* represent only 11% and 6%, respectively (Supplementary Fig. S3). Generally, the relative abundance of *Proteobacteria* ranges from 53% to 80% of the total effective bacterial sequence, as revealed by Miseq high-throughput sequencing, which indicates the abnormal enrichment of hydrocarbon-degrading bacteria in sediment. As shown in Fig. 3, *Oceanospirillales* were the main constituents at the Order level from D0 to D15, likely due to their versatile abilities for motility, chemotaxis and aliphatic hydrocarbon degradation, as revealed in the first stage of the Deepwater oil spill^29,30^. A significant shift occurred at D22, as the *Chromatiales* replaced the *Oceanospirillales* as the dominant OTU. Other changes during the course of natural biodegradation include an increasing proportion of *Pseudomonadales* and *Xanthomonadales* and a decrease in *Alteromonadales*. However, *Rhodobacterales* and *Sphingomonadales*, as effective PAH degraders in soils and sediments, were widely detected in the sediment^31,32^. A bloom of both *Alcanivorax* and *Dietzia* was observed at D0 and D3 and can completely cover 35%-40% of the effective reads at the genus level (Fig. 4). The relative abundances of RDH and *alk*B genes were also analyzed. Both genes reach their high abundance at early stage (D0 and D3) and then show an increasing trend after tidal action at D12 and D32 (Fig. 5C and D).

## Discussion

It is well known that the response of the microbial community to the oil washed ashore plays a key role in determining the magnitude and degree of degradation of residues before they penetrate into the deep layer. A large body of publications has concluded that subsurface residue oil is difficult to degrade and can persist for decades^1,2,32,33^. In this monitoring site, high-throughput sequence analysis suggests that *Proteobacteria* are dominant in the whole community in oil-polluted gravel beach sediment, with a relative abundance up to 80% (Fig. 3 and Supplementary Fig. S2). The relatively high abundance of *Alphaproteobacteria* (15.4%-28.09%) and *Gammaproteobacteria* (36.14%-51.59%) in the sediment correlates (Spearman’s rank correlation) well with decreasing concentrations of aliphatic and aromatic hydrocarbons (Supplementary Tab. S1 and Fig. S4), which is strongly indicative of *in situ* ongoing biodegradation. More specifically, Student’s t-test of significance was performed between the abundance of *Oceanospirillales*, *Actinomycetales*, *Rhodobacterales*, *Alteromonadales*, *Alcanivorax* and the concentration of Trmn, Phe, resins and asphaltenes over time (Supplementary Fig. S4), results suggested these microbial taxa played significant role in hydrocarbon degradation. The results suggest that the beach microbial community here has a wide range of substrates and exhibits a diversity of hydrocarbon-degraders, with great potential for a complex metabolic network^34^.

Interestingly, *Alcanivorax* and *Dietzia* were significantly enriched in sediment, each representing up to 20% of the total reads at the genus level (Fig. 4). The high relative abundance of *Alcanivorax* and *Dietzia* here possibly results from the low biodiversity of other oil-degrading bacteria due to natural features of the gravel beach^35^. As shown in Fig. 4, the relative abundance of *Alcanivorax* at the first four sampling points (D0, D3, D12, and D15) ranges from 15.56% to 22.15% of the total OTUs. Previous studies showed that *Alcanivorax* occurs in small amounts in unpolluted areas and only becomes dominant upon the intrusion of oil and oil-associated components^35,36^, although it is widely distributed in the Atlantic Ocean and Pacific Ocean^37^. Therefore, the rapid assembly of *Alcanivorax* here can be regarded as direct evidence for the robust response of an indigenous bacterial community to spilled oil. Generally, enrichment of *Alcanivorax* means that microbial communities harbor a high capability for hydrocarbon degradation, which was also observed during the Deepwater Horizon oil spill by Kostka *et al*.^18^, where a bloom of *Alcanivorax* in a sandy beach reached up to 13.3% of the entire community several months after the accident. *Dietzia*, another alkane degrader, can provide bio-surfactants^38^ and bio-demulsifiers^39^, which increase oil bioavailability by dispersing patched residues into small droplets and provide attachment for hydrocarbon degraders. This suggests that the high relative abundance of *Alcanivorax* and *Dietzia* in beach sediment can result in mutually beneficial cooperation that accounts for the most effective biodegradation of residue oil, as well as the development of an active hydrocarbon degrading system.

Tidal action, as typical shoreline energy, has been recognized since the 1970–80 s as having an important role in accelerating oil degradation in the natural attenuation process by increasing the surface area of patched residues and nutrient exchange with fresh water^13,40^. However, it is still ambiguous whether tidal action during an oil spill, i.e., periodic oil input into beach sediments, can affect the development and/or dynamics of hydrocarbon-degrading consortia. In this study, three periodic tidal actions were recorded in the full experimental time sequence (Fig. 5). The first two tidal actions transported significant amounts of floating oil, while the third almost transported negligible oil to the gravel beach because most of the floating oil was removed in the two months following the oil spill (Fig. 5). As shown in Fig. 5A and B, the concentrations of all compounds studied over time were strongly affected by periodic tidal action. The measured chemical components showed rapid degradation followed by increased concentrations at D12, indicative of the first tidal action. Subsequently, a new round of biodegradation occurred from D15 to D22, again with the rapid depletion of those components until the arrival of another wave action. The concentration of the measured chemical components remained at a low level after the third wave at D32, suggesting that less lingering oil was brought to the beach, consistent with the clearance of floating oil in the accident area after two months.

Previous studies showed that even mild wave action can disperse floating oil into tiny droplets (<70 μm)^41^, thus acting as a natural dispersant to improve the bioavailability of the residue oil and making oil drops easier for microbes to access, transport and degrade^2^. The high degradation efficiency of residue oil after every wave action (*e*.*g*., D0 to D3, D12 to D15) likely indicates that the beach microbial community can easily access the oil components, which may be partly due to the tidal dispersal effect. *Oceanospirillales* and *Alteromonadales* are the predominant hydrocarbon-degrading bacteria (order-level) at the initial sampling point (D0) and continue to maintain a similar structure from D0 to D12 (Fig. 3), coinciding with the rapid degradation of residual oil. The development of *Oceanospirillales* was confirmed at the first stage of the Deepwater oil spill^30,42^. *Altermonas*, a genus of *Altermonadales*, was widely reported to have a broad range of degradation capacity, and it was reported as the dominant PAHs degrader in the Yellow Sea^43^. Both *Altermonadales* and *Oceanospirillales* were confirmed as important short-chain hydrocarbon degraders^5,29,33^. After D22, the relative abundance of *Oceanospirillales* and *Alteromonadales* decreases significantly due to depletion of aliphatic and aromatic hydrocarbons and the enrichment of resins and asphaltenes (Table 1). The dominant taxa are replaced by *Chromatiales*, *Rhizobiales* and *Xanthomonadales*, indicating functional shifts in microbial groups from alkanes and low molecular-weight aromatic hydrocarbon degraders to specialized high molecular-weight hydrocarbon degraders. At the genus level, after the first wave action, the relative abundance of *Dietzia* decreased from 20% to 0.03%, whereas *Alcanivorax* showed a slight decrease and remained at 15% on D12. When the second wave arrived at D22, *Alcanivorax* decreased drastically to below 0.03%, whereas *Dietzia* also remained low. Both began to gradually recover after D22 and remained at 15% (Fig. 4). This substantial shift over time coincided with relative quantities of lighter hydrocarbon fractions and indicated that rapid and effective hydrocarbon degradation occurred at an early stage of the oil spill (D0-D22). Calculated from the concentration of isoprenoids from Table 1, the relative degradation level of aliphatic hydrocarbon at D22 can reach up to 96%, suggesting that the supply of hydrocarbon acts as the determining factor to regulate microbial composition. This microbial succession pattern was previously suggested from a 2D model that tracked circulation, bacterial abundance, metabolic rate and other data during the Deepwater Horizon oil spill^17^. In this study, the phylogenetic shift in the microbial community keeps pace well with pulsed oil input induced by tidal action, and the corresponding genes involved in hydrocarbon degradation provided further functional information on the response of the indigenous microbial community to residue oil. As shown in Fig. 5C and D, the abundance of the RDH and *alk*B genes is fully consistent with the concentration of bioavailable hydrocarbons and suggests a rapid aerobic degradation response to the massive input of hydrocarbons. Significant enrichment occurred after the first two tidal actions, possibly due to the supplement of floating oil, which caused a new bloom of hydrocarbon degraders. The third tidal action at D32 did not produce a significant increase in the RDH and *alk*B genes because less bioavailable component remained in seawater that can be transported to the shore. The results demonstrate a clear microbial succession pattern, including both a phylogenetic shift in the hydrocarbon-degrading bacteria community and the dynamic of functional genes accompanied by tidal action during the sampling campaign.

Although there was an excellent correlation between the hydrocarbon-degrading consortia dynamic and pulsed oil input induced by tidal action, which strongly indicates a mass dependence on the development of the microbial community, the “autoinoculation effect” hypothesis is still uncertain because floating oil can also bring hydrocarbon-degrading bacteria to the beach ecosystem by tidal action and eventually have a positive effect on the development of the indigenous microbial community. For example, *Oceanospirillales* was reported as planktonic bacteria during the Deepwater Horizon oil spill^29^. Miseq high-throughput sequencing results show that *Oceanospirillales* dominated the OTUs during the sampling campaign and accounted for more than 20% of the OTUs at the order level (Fig. 3), suggesting that the high abundance of *Oceanospirillales* in beach sediment likely results from tidal action; the floating oil itself sustained a microbial seed bank of oil-degrading bacteria. However, the Dalian is an industrial city for oil refining and port transportation, and the coastal sediment has a historical accumulation of oil contaminations^21^. Frequent tanker shipping would have an acclimatizing effect that reinforces the adaptation of indigenous microbes for greater hydrocarbon-degrading capacity under the *in situ* conditions^5^. After the Xingang oil spill, more than 50 strains of cultivable oil-degrading bacteria, including *Alcanivorax* and *Marinobacter*, were screened in previous studies from the coastal sediment^5,44^, and 12 bacterial strains were further isolated for the first time as oil-degrading bacteria, suggesting that Dalian Bay possibly harbors a previously undetected microbial “seed bank” of hydrocarbon-degrading consortia. After the oil spill, the indigenous aerobic microbial communities were activated by floating oil washed ashore, and typical environment-disturbance niches were developed to select and sustain hydrocarbon degrading bacteria, which are characterized by a diverse and abundant oil-degrading community, as revealed by Miseq high-throughput sequencing (Fig. 3). In addition to the natural attenuation process, the microbial structure is dynamically regulated by bioavailability and the supply of hydrocarbon components (Fig. 5). Typically, the decay of an *Alcanivorax* bloom correlates well with the rapid degradation of related chemical components (Fig. 4, Table 1). Subsequent stimulation by floating oil, followed by a continued increase in *Alcanivorax* and *Dietzia* (Fig. 4), is in agreement with the physical metabolic model suggested by Valentine *et al*.^17^. Although oil-degrading bacteria brought by floating oil had a positive effect on the development of a microbial community at the beach, the indigenous microbial community still plays a major role in the succession of the microbial community, as revealed by the blooming of the microbial community and degrading genes concurrent with pulsed oil input that was induced by tidal action. If this is mainly induced by floating oil-associated bacteria, this synchronization cannot be expected due to its stable carbon source within floating oil. Therefore, we consider that regular inputs of the floating oil act as a trigger to stimulate the “seed bank” of hydrocarbon-degrading bacteria and have an “autoinoculation effect”, leading to the continued blooming of hydrocarbon-degrading consortia, while spreading the oil broader and deeper. In other words, repeated oil input may exert a deterministic effect to influence species assembly into a local community, resulting in the continued blooming of *Alcanivorax*, *Dietzia*, *Marinobacter*, *Sphingomonadales*, *Rhodobacterales* and *Alteromonadales* (Fig. 3 and Fig. 4).

The last question concerns the nutrient supply for sustaining hydrocarbon-degrading bacteria at the beach. Although beach sediment is traditionally characterized as an oligotrophic environment that limits the growth of native microbes, and nitrogen addition was widely used (as in the Exxon Valdez oil spill^45^ and Prestige oil spills^46^), the Dalian Bay could be an exception due to severe eutrophication conditions since the 1990s^47–49^. The concentrations of dissolved inorganic nitrogen (DIN) and dissolved inorganic phosphorus (DIP) reached 24.60 ± 15.35 μmol/L and 14.96 ± 4.09 μmol/L, respectively^49^. This suggests that tidal action can transport sufficient nutrients via water waves to meet microbial growth and metabolic demands, and further promote a robust, rapid response to spilled oil in the early stage.

In conclusion, our study strongly suggests that tidal action actually acted as a deterministic process mediating the microbial community’s ecological succession in the beach, leading to the continued blooming of known oil-degrading bacteria by stimulating the well-developed “seed bank.” This is in exact agreement with the “autoinoculation effect” hypothesis. Subsequently, the original microbial succession process was interrupted by tidal action, which then initiated a deterministic succession process, as indicated by the long duration of abundant *Alcanivorax* and *Dietzia*.

## Methods

### Site description and background

The Dalian Xingang oil port is located in the southern part of the Yellow Sea, Liaoning Province, North China. It is one of the busiest cargo ports for crude oil transportation in China. The gravel beach polluted by lingering oil was maintained to monitor the natural attenuation process. This area, found 34 days after the spill accident, is 25 km from the spill site and located within the heavily polluted coastal area^3^. It was maintained for 52 days for time-sequence sampling under natural conditions, labeled D0, D3, D12, D15, D22, D33, D42 and D52. This gravel beach was periodically affected by tidal action without anthropogenic disturbance and therefore had frequent fresh oil input until clearance of the floating oil in the accident area after two months. Periods of great tidal action when floating oil washed ashore were recorded, including peak tides.

### Chemical analysis of residue oils

Oil-polluted sands were collected in a time sequence and residue oil was recovered by Soxhlet extraction using dichloromethane (DCM)–methanol mixtures (97:3 V:V), followed by purification with anhydrous Na_2_SO_4_. Asphaltene was removed from the residue oil by precipitation with petroleum ether, followed by filtration. The maltene (de-asphaltene) was separated into aliphatics, aromatics and resins by column chromatography with activated silica gel and alumina as the stationary phases. The aliphatic and aromatic fractions were analyzed by an Agilent 5973 mass spectrometer coupled to an Agilent 7890 gas chromatograph (Agilent Technologies, USA). Internal standards (*n*-C_24_D_50_ and phenanthrene-d10) were added before the measurements, and the SIM model was used to quantify the concentrations of interest: m/z 183 for isoprenoids, m/z 170 for trimethylnaphthalenes, m/z 178 for phenanthrene, and m/z 192 for methylphenanthrenes.

### Total DNA extraction, 454 and Miseq sequencing

Genomic DNA was extracted using a PowerSoil® DNA isolation kit (MoBio Laboratories, Carlsbad, CA, USA), using the manufacturer’s protocol. The extraction of DNA was examined by gel electrophoresis and quantified with a NanoDrop ND-2000 spectrophotometer (NanoDrop Technologies, Wilmington, DE, USA). All samples were sent for illumina Miseq sequencing, and one sample (D22) was sent for GS FLX Titanium amplicon pyrosequencing (Roche 454) to obtain deeper insight into the community. Community analysis was conducted using the microbial ecology software program QIIME (http://qiime.org/)^50^. The raw sequences were deposited at the National Center for Biotechnology Information (NCBI) Short Read Archive database under the accession number PRJNA289049.

### Quantitative real-time PCR assays

All real-time qPCR assays were performed in triplicate on a Qiagen Rotor Gene qPCR system (Qiagen, Germany). The 16 S rRNA gene amplified by the total bacterial primers was used as a housekeeping gene to target an approximately 180-bp region. The ring-hydroxylating dioxygenase (RDH) gene^51^ and alkane 1-monooxygenasegene (*alk*B) gene^18^ were chosen to quantify the polycyclic aromatic hydrocarbon- (PAH-) and alkane-degrading functional groups. PCR amplification was performed in a total volume of 50 μL containing 25 μL of FastStart Universal SYBR Green Master (ROX, Germany), 1 μL of DNA template and 0.5 μM of each primer. The thermocycling steps for qPCR amplification were as follows: 95 °C for 10 min, followed by 40 cycles of 95 °C for 15 s and 60 °C for 60 s. The PCR products were visualized and checked by agarose (1%) gel electrophoresis in the presence of DL2000 markers (Takara, Japan). The relative abundance of the functional group was normalized by targeting the gene expression against the 16 S rRNA of each genomic DNA sample using the 2^_CT^ method^52^.

### FT-ICR MS analysis

The crude oils were dissolved in toluene to produce a 10 mg/ml solution for ESI FT-ICR MS analysis. A total of 20 μL of solution was further diluted with 1 ml of toluene:methanol (1:1, v-v) solution; 15 μL of ammonium hydroxide solution (28%) was added to facilitate deprotonation of the acids and neutral nitrogen compounds to yield [M-H]^−^ ions. The crude oil and its fractions were analyzed using a Bruker apex-ultra FT-ICR mass spectrometer equipped with a 9.4 T superconducting magnet. Sample solutions were infused via an Apollo II electrospray source at 180 μL/h with a syringe pump. Typical operating conditions for negative-ion formation were emitter voltage, 4.0 kV; capillary column introduce voltage, 4.5 kV; and capillary column end voltage, −320 V. Ions accumulated for 0.1 s in a hexapole with 2.4 V of direct current (DC) voltage and 400 Vp-p (volts peak to peak) of radio-frequency (RF) amplitude. The optimized mass for Q1 was m/z 250. An argon-filled hexapole collision pool was operated at 5 MHz and 400 Vp-p of RF amplitude, in which ions accumulated for 0.4 s. The extraction period for ions from the hexapole to the ICR cell was 1.2 ms. The mass spectrometer was calibrated using sodium formate. Mass peaks with a relative abundance greater than 6 times the standard deviation of the baseline noise level were exported to a spreadsheet. Data analysis was performed using custom software, which has been described elsewhere^53^. Compounds with the same heteroatom class and its isotopes with different values by DBE and carbon number were searched within a set ± 0.001 Kendrick mass defect (KMD) tolerance^54^.

### Statistical analysis

Statistical calculations were performed using the R statistical platform^55^ (R version 3.3.3) with the package corrplot (https://github.com/taiyun/corrplot) for correlation analyses and visualization.

## Electronic supplementary material

Supporting Information
