# Supplementary material for: Periodically spilled-oil input as a trigger to stimulate the development of hydrocarbon-degrading consortia in a beach ecosystem
Source: Sci Rep. 2017 Sep 29;7:12446. doi: 10.1038/s41598-017-12820-7 (PMC5622073; doi:10.1038/s41598-017-12820-7)
Supplement: Supplementary file 1 — Supporting Information [file 41598_2017_12820_MOESM1_ESM.doc]

**Scientific Reports**

**Supporting Information**

**Periodically spilled-oil input as a trigger to stimulate the development of hydrocarbon-degrading consortia in a beach ecosystem**

**Kai Zhang^1^, Yongge Sun^1*^, Zhisong Cui^2^, Di Yu^1^, Li Zheng^2^ , Peng Liu^1,3,4^, Zhenmei Lv^5^**

**^1^ Environmental and Biogeochemical Institute (eBig), School of Earth Science, Zhejiang University, Hangzhou, Zhejiang 310027, China.**

**^2^ The First Institute of Oceanography, SOA, Qingdao, Shandong 266061, China.**

**^3^ Wuxi Research Institute of Petroleum Geology, SINOPEC, Wuxi, Jiangsu 214126, China.**

**^4^ State Key Laboratory of Shale Oil and Gas Enrichment Mechanisms and Effective Development, Wuxi, Jiangsu 214126, China.**

**^5^ School of Life Science, Zhejiang University, Hangzhou, Zhejiang 310058, China.**

*** Corresponding author:** [**ygsun@zju.edu.cn**](mailto:ygsun@zju.edu.cn)**.**

Figures and Tables

Figure captions

Figure S1. The distributions of aliphatic hydrocarbons, steranes and terpanes, showing dynamics of alkanes and biomarkers in residue oil upon time sequence.

Figure S2. Relative abundance of heteroatom class species in beach oil residues derived from negative-ion ESI FT-ICR mass spectra.

Figure S3. Microbial community structure (Phylum level) as revealed by 454 pyrosequence in sediment samples D22.

Figure S4. Correlation matrix based on Spearman’s rank correlation. The color scale visualizes significant correlations (Spearman’s ρ) between the relative abundance of microbial taxa and oil compounds, all p values were shown in the figure.

Table captions

Table S1. Compound-grouped fractions (%) in the residue oils during the sample campaign.


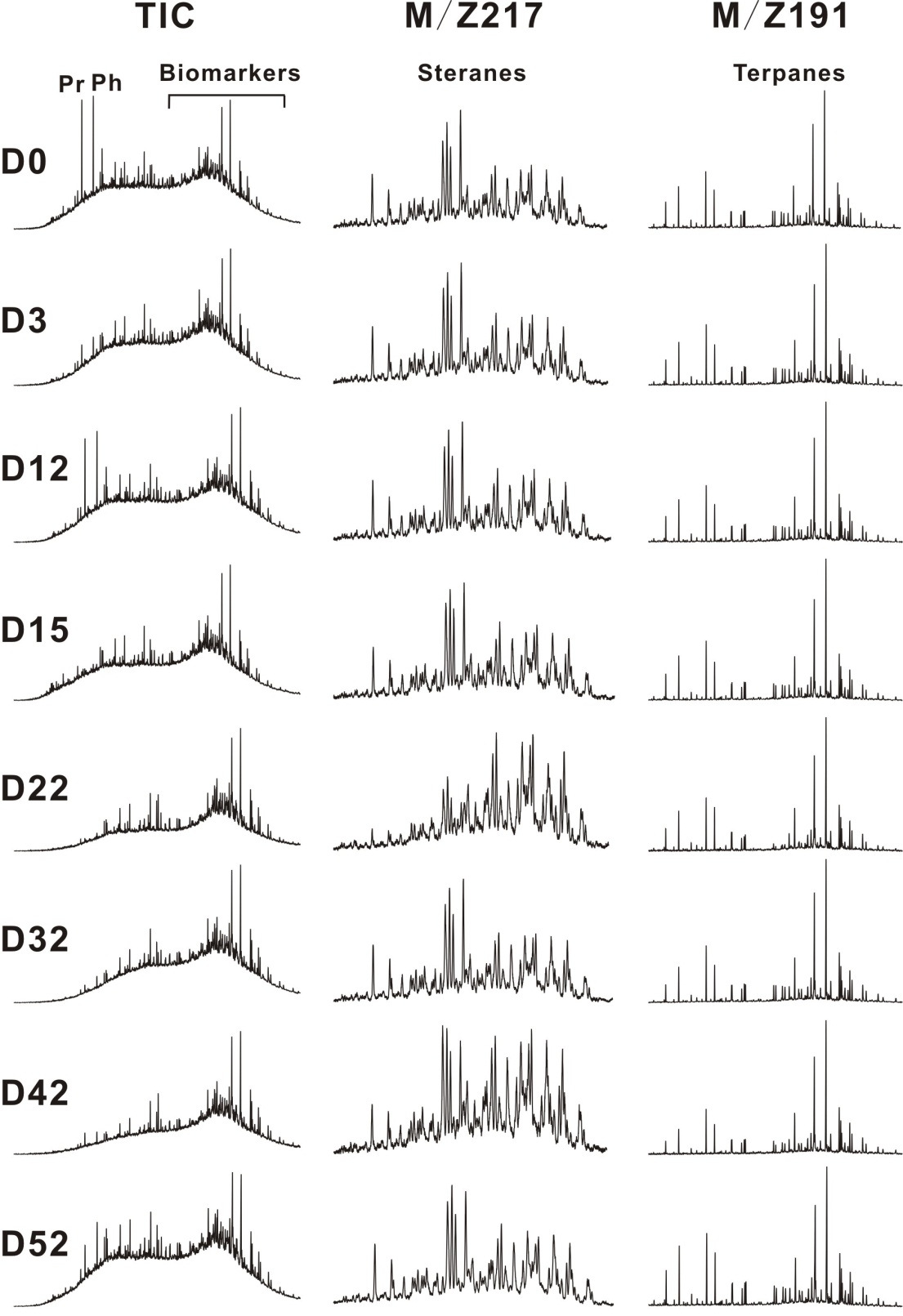


Figure S1.

**
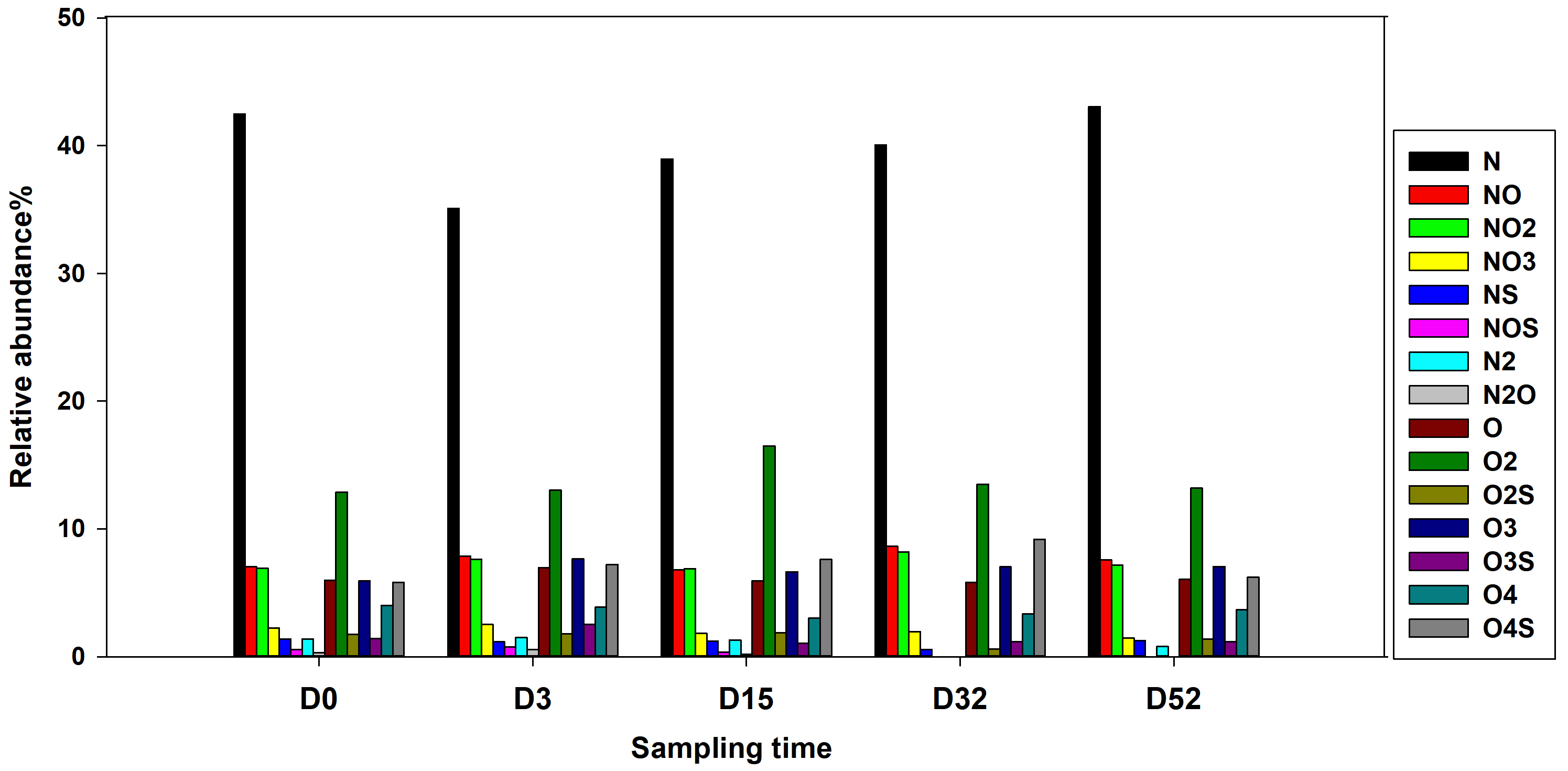
**

Figure S2


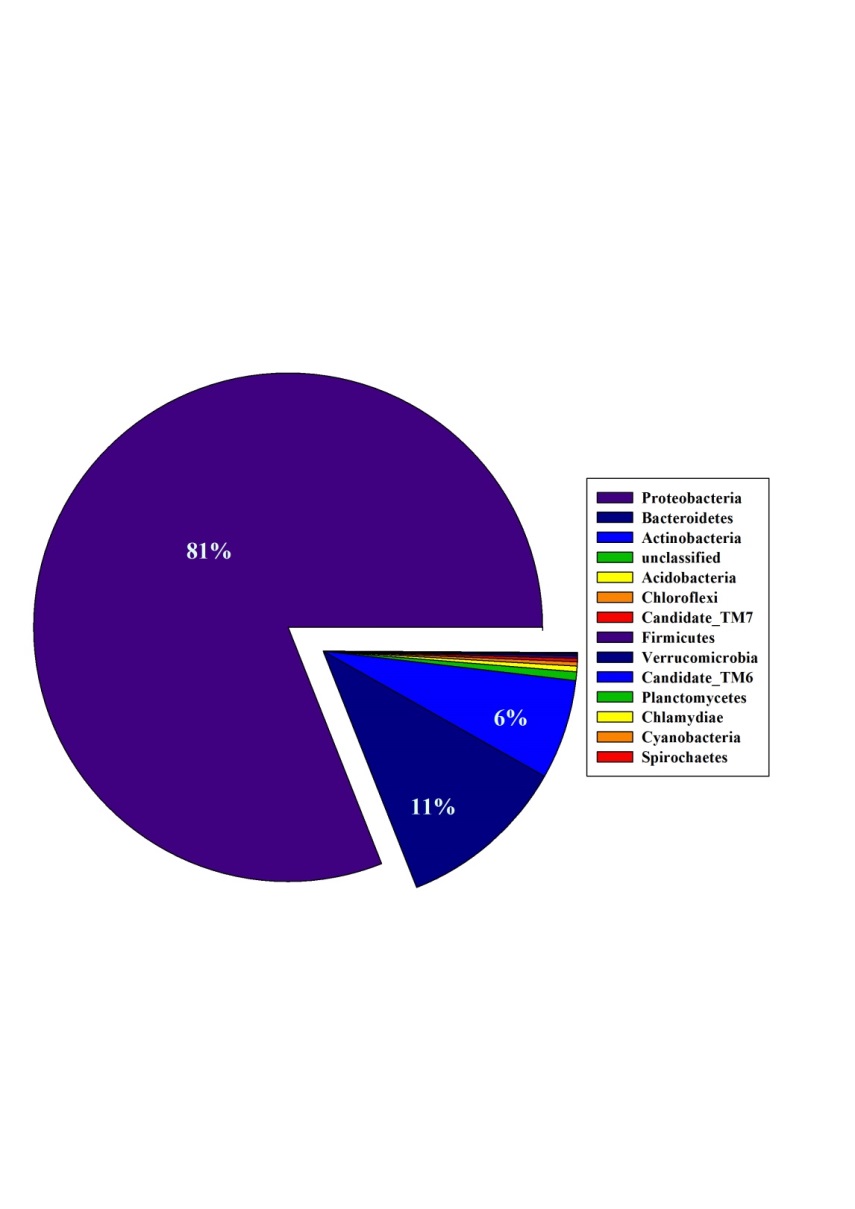


Figure S3


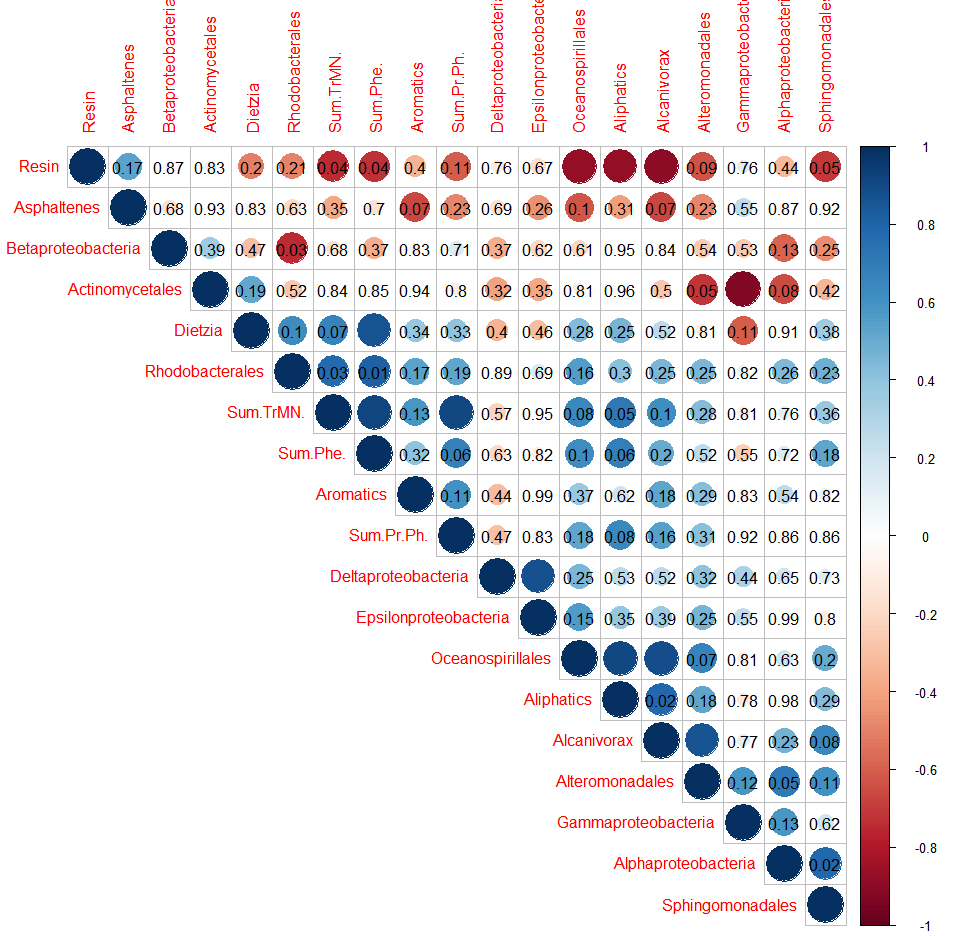


Figure S4

Table S1

| Samples |  | Relative abundance (%) | | |
| --- | --- | --- | --- | --- |
|  | Aliphatics | Aromatics | Resin | Asphaltenes |
| D0 | 32.89 | 28.95 | 17.11 | 14.91 |
| D3 | 28.79 | 26.63 | 16.56 | 18.42 |
| D12 | 28.69 | 27.69 | 16.93 | 15.54 |
| D15 | 28.01 | 23.83 | 18.92 | 15.48 |
| D22 | 14.57 | 27.15 | 22.52 | 21.19 |
| D32 | 24.69 | 19.26 | 20.00 | 26.17 |
| D42 | 20 | 24.44 | 19.26 | 15.56 |
| D52 | 26.9 | 25.89 | 19.04 | 16.75 |
